# The “thinking system” in a new school concept: A rhythmic teaching approach in physical education to develop creativity

**DOI:** 10.1371/journal.pone.0301858

**Published:** 2024-04-16

**Authors:** Marta Rigon, Pietro Luigi Invernizzi, Gabriele Signorini, Athos Trecroci, Raffaele Scurati, Damiano Formenti, Dario Colella, Andrea Bosio, Domenico Cherubini

**Affiliations:** 1 Department of Biomedical Sciences for Health, Università degli Studi di Milano, Milano, Italy; 2 UCAM Catholic University of Murcia, Murcia, Spain; 3 Department of Biotechnology and Life Sciences (DBSV), University of Insubria, Varese, Italy; 4 Department of Biological and Environmental Sciences and Technologies, University of Salento, Lecce, Italy; 5 Human Performance Laboratory, Mapei Sport, Olgiate Olona, Italy; Damghan University, ISLAMIC REPUBLIC OF IRAN

## Abstract

System Thinking is an actual construct supported by several scientific evidence that offer a perspective on how phenomena relate. Rhythm methodology, teaching-learning, and enjoyment in physical education are the main system elements we hypothesize interacting closely to determine direct or mediated effects on motor creativity and rhythmic perceptive capacity. Seventy-six elementary and middle school students (8.9 ± 2.1 years) were randomly assigned to two groups: a) an intervention group that received a physical education lesson based on rhythmic methodology and b) a control group that received conventional lessons without specific rhythmic interventions. Participants were engaged in eight physical education lessons lasting one hour each for eight weeks. Tests and questionnaires were administered before and after the intervention to evaluate motor creativity, rhythmic perception capacity, self-perception and enjoyment. Two lessons were randomly analyzed to identify the teaching style and motor content (moderate and vigorous activity). The main results revealed direct effects on the intervention group’s motor creativity (p = 0.001) and its rhythmic perception capacity (p = 0.02). Furthermore, enjoyment mediated the effects of the intervention on motor creativity (p = 0.01). Finally, the results have shown that self-perception does not mediate the effect of rhythmic intervention group on motor creativity and rhythmic perceptive capacity (p > 0.05). A rhythmic methodology proposed by specific multi-teaching styles can involve children and young people in an enjoyable activity with more moderate to vigorous physical activity.

## Introduction

### Thinking system and scholastic vision

A thinking system approach considers complexity as an intrinsic element of every change process, and this can be extended even to a formative path. This topic addresses a growing interest related to the community of practice and the connection with other elements that can be functional in managing educational policy in real contexts. The thinking system approach encompasses emerging ideas and methods that promote the exploration of the most comprehensive framework, encouraging one to look at the bigger picture [1–3]. Thinking system can be traced back to three keywords: interrelation, as the connection between the elements of the system; multiple vision, as an approach to the issue that considers different viewpoints and perspectives (as qualitative and quantitative research with the use of mediators and moderators); boundaries, which delimited the interconnected elements and what is external to this system of interests. Literature has already shown a multi-faceted integrated system approach [4–6].

The framework proposed in a previous study is an example of how the formative system results from a complex relationship of many integrated domains, with different points of view and some elements found inside and outside the system of interest [6].

This way, to embrace an integrated and complex vision, the school is increasingly oriented towards a transdisciplinary approach that reflects the thinking system. The interrelation of several disciplines promotes a learning system based on a multiple vision, in which each discipline while maintaining its identity and characterizing elements, i.e., its boundaries, can also lead to other subjects enriching the learning process (i.e., music and physical education to improve on rhythm and motor creativity). Disciplines should not be presented as territories to be protected by defining rigid boundaries but as interpretive keys available for every possible use. Complex problems require different disciplinary viewpoints to dialogue with each other and to pay attention to the border and hinge zones between disciplines [7].

### Rhythmic training and systemic view in scholastic context

The ability to sequence is very important in children’s learning process. Rhythm-based practice in school settings enables children to acquire good temporal and rhythmic perception, which are essential for learning reading, writing, and language skills [8]. In a systemic view, the interrelation of music and physical education, through rhythmic perception and motor creativity improvement, can enforce the learning of reading, writing, and language skills in a multiple-vision concept [9–12]. In music, rhythm is the most important structural element. The music domain is organized into temporally structured sequences. It is composed of rhythmic structures that underlie an important organization of temporal successions [13]. Its metric organization is similar to linguistic syntax. Sensory-motor integration, motor control, and action planning can be analyzed through rhythmic syntax [14]. It is common to see how movement can be easily integrated within a musical environment through dancing, tapping, or nodding one’s head to follow the rhythm. In more complex situations, two or more people interact during musical activity, playing and dancing together [15], and in social contexts, individuals act concurrently. Rhythm denotes a quality aspect of movement. More specifically, rhythmic movement in physical education considers a structured sequential form to utilize for favoring the acquisition of motor skills or expressions movements as manifestations of mental, social, and emotional responses [16].

### Creativity in scholastic thinking system

Creativity must be considered a process incorporated in the cultural sphere of reference [17].

Creativity represents an act, an idea, that is produced in a particular field or that, starting from an existing field, generates a new one. Symbolic elements and specific notation systems mediate the knowledge expressed in the creative act. Each domain follows specific and rigid rules that limit its nature, modes of expression, and accessibility [18]. Creativity understood as a system must consider three components [18]:

1. The domain, made up of the specific symbolic rules and procedures that make up its structure. The domain referred to in this study consists of scholastic physical education and, with a finer concept analysis, in motor creativity.
2. The field, which refers to "the custodians" of the domain through which the possibility of including a new idea and didactic path is considered. The field in the school environment directly involves the teachers of the disciplines
3. People directly involved in the didactic-creative action (schoolchildren), and who, using the symbols of a domain, learn to express and identify a new pattern

Creativity in a specific domain has boundaries that determine what content can be included and what should be excluded. This way, transversality is a modality that can be used to grant new skills through the opening to accessible and related domains.

The efficacy of the choice of a related domain must be based on the criteria of clarity and simplicity of the new structure, its accessibility, and its possible recognizable presence in the main domain.

Transversal and transferable skills through the operational dimension of doing (as in the context of connection between rhythm and movement) can include new processes of critical thinking forming the basis of the creative domain of PE. These processes involved motricity and stimulated a new type of reflective and internalized learning that plays an essential role in self-construction, in which the student is an actor in his or her skills and knowledge acquisition [19].

Developing creativity in children is important to let them express themselves through the art of music and body expression. The student’s proficiency in these multi-perspective skills can significantly impact their attitude, strategies’ effectiveness, interpersonal interactions, and ability to gather and utilize feedback for adapting and improving actions across the considered domains [19]. In this regard, it becomes important that the context provides many possibilities for acquiring, memorizing, internalizing, and reproducing the stimuli of the different integrated domains. The school must develop integrated educational action to foster connections between relational domains and domains involving teachers who, in a transversal approach, follow a common path of intervention [19].

In this process, physical education and rhythm are the elements of the system that are considered within and beyond the interest in developing critical thinking processes. In particular, rhythm is conceived as a point in a system that can produce broad changes in motor creativity (leverage point) [20].

### Added value by considering a comprehensive set of factors influencing motor creativity (understanding the complex drivers that can foster learning)

#### Factor 1: Teacher and motor creativity

Although the students occupy a central position in their learning process, the teacher also plays a crucial role in success. The teacher acts as an artist who has to shape his opera, referring to the context. He or she must create proper transdisciplinary pathways to achieve the transversal competencies. The teacher plays a key role in this complex system: through proper teaching practice, he/she can influence the students to learn and improve in a specific field. School autonomy [21] lets the teacher find strategies peculiar to the context to ensure their attainment of competencies at the end of the education cycle. The teacher is responsible for employing an integration teaching style related to the context [7]. Several studies highlight a relationship between the teacher’s ability to vary the use of different teaching styles and the involvement of cognitive, motivational, motor, and emotional aspects essential for maintaining an interest in developing creative-motor skills [22, 23].

Not surprisingly, an integration of several teaching styles allows for better creative thinking outcomes than a classical linear approach. Such an approach stimulates the student to find more personal solutions through continuous exploration of different and creative motor behaviors and flexible use of attention to solve environmental problems. Furthermore, through teaching styles that stimulate a maieutic and collaborative process, he or she can help the student develop his or her soft skills, which are indispensable for building the relationship with the teacher himself or herself and on which the outcome of the learning process depends [24, 25]. The teacher’s task is to encourage motor activity within the educational process.

He or she can successfully achieve this goal through rhythm-based practice, offering age-appropriate qualitative and quantitative stimuli. Music, by its nature (temporal scansion and succession, structure, rhythm, sequencing), facilitates and makes the approach to motor practice almost spontaneous. A multi-teaching approach involving music, physical education, and creativity could enhance the learning process [26].

The relationship between music, movement, and creativity can be explained by the intervention of the primary motor cortex M1, which appears to mediate the potential for creative motor action associated with a pre-planned creative idea. The basis for a creative idea may form in brain areas associated with higher-order creative processes and then flow in part through M1 and be realized as a creative motor action [12].

In addition to the teacher’s ability to establish relationships with his or her students, the ability to elicit enjoyment also plays a crucial role in the learning process [27].

#### Factor 2: Enjoyment and motor creativity

Enjoyment is a positive psychological effect that reflects happiness, pleasure, enthusiasm, liking, excitement, and fun [28]. In physical activity, enjoyment represents a positive attitude toward practice and is one of the most important aspects of motor development and sports participation [29].

Adequate levels of enjoyment and self-perception of physical fitness increase motivation, engagement, and participation in physical activity [30]. Pleasant and fun motor experiences in varied contexts are crucial for acquiring adequate bodily self-awareness in children and young people [31].

#### Factor 3: Motor creativity and self-perception

Assiduous participation in physical practice makes it possible to acquire and consolidate new motor patterns and increase motor competence in the long run. Children with a high level of motor competence are more likely to have an active lifestyle that positively influences physical and mental well-being [32]. Motor competence relies on its specific and highest manifestation in motor creativity, a perceptual ability that favors the development of new motor patterns aimed at solving problems or as body expression of ideas and emotions [33]. Motor creativity enables the production of motor movements and gestures to solve specific motor tasks or express emotion physically [33, 34].

Compared to motor competence, motor creativity develops more through exploratory teaching in an environment with well-adapted constraints, where kinesthetic variety is supported and where everyone is free to experiment through movement [34, 35]. Varied and positive kinesthetics and motor experiences improve the self-perception of children and young people by promoting the awareness of successfully acquiring different motor skills [36]. This capacity for self-perception is linked to the cognitive, motor, and social resources that the subject can mobilize in the different contexts in which he/she must produce motor skills [32]. The quality of experience provided by the teacher through effective teaching processes increases the positive self-perception capacity of young people [37, 38].

### Research questions

Two research questions can be posed considering the following premises and the complexity of the thinking system approach in the school system. Does a skilled teacher using rhythm-based activities and appropriate challenges help students become more creative in their movements and better at recognizing rhythms? Also, how does the enjoyment of the activities, feeling competent, and age influence this learning process?

## Materials and methods

### Participants

Seventy-six elementary and middle school students were voluntarily recruited (from the 12th to the 19th of September 2022) for our study. Thirty-two students were in the second year of elementary school (2^nd^ elementary school), twenty were in the fourth year of elementary school (4^th^ elementary school), and twenty-two were in the third year of middle school (3^rd^ middle school). Thirty-six participants were males, and forty participants were females. The students were divided into an experimental (section A) and a control group (section B), utilizing a class-randomized procedure. In this study, the experimental group underwent a rhythm-based intervention (RI), while the control group did not receive any rhythm-based intervention (NI). Participants’ demographics are shown in Table 1. All participants and their parents were informed about the purpose and experimental protocol of the study. Parents or legal guardians provided written informed consent before the investigation. Following the Declaration of Helsinki, the study was approved by the Ethics Committee of the local University (approval number 18/22).

**Table 1:** Participants’ demographics.

Group	Male	Female	n	Age (years)	Height (m)	Weight (kg)	BMI (kg/m^2^)
RI	21	22	43	9.02 ± 2.03	1.38 ± 0.16	35.00 ± 12.53	17.53 ± 2.87
NI	15	18	33	8.84 ± 2.22	1.38 ± 0.17	34.63 ± 13.3	17.60 ± 2.94

Data are mean ± SD; RI = Rhythm Intervention; NI = No-Rhythm Intervention. BMI = Body mass index.

### Measures

#### Rhytmic perceptive capacity

Mira Stambak test was employed to assess the rhythmic perceptive capacity. Stambak’s test evaluates spatiotemporal structure by testing the rhythmic ability on multiple levels as a replication of temporal structures: the evaluator plays twenty rhythmic sequences in which each circle corresponds to a beat, and each space corresponds to silence. The child had to reproduce it respecting the beating and resting time; symbolization of spatial: the evaluator shows ten rhythmic structures one by one. After showing a structure for a few seconds, the examiner hid it from the participant. The participant had to memorize the structure and reproduce graphically each sequence; symbolization of temporal structures: the examiner reproduces the rhythmic structures by tapping. After listening, the participant had to write the sequence respecting circles and spaces. In each task, after two consecutive incorrect sequences, the test ends. A point is obtained when each successful structure is performed, and, in the end, the points obtained for each level are added [39]. Since 1951, Stambak’s test has been used in different fields and research demonstrating its validity and reliability [6, 39, 40].

#### Motor creativity

Divergent Movement Ability (DMA) test was performed to investigate motor creativity in school-aged children. The DMA’s validity and reliability were previously established [41]. No warm-up was done before performing the test. DMA measures the fluency and flexibility dimensions of creativity of three dimensions of movement skills: locomotor, for which an individual is asked to use the tools provided (e.g., two mattresses of different sizes, four cones arranged diagonally, a suspended rope, and a hoop supported by three cones) stimulating them to create as many movements as possible; stability, for which an individual is free to take on different figures (with their body parts) within an area with a 45.5 cm high bench placed in the middle; manipulation, for which an individual handles a ball (23 cm diameter) within an area (3 m × 4.5 m) delimitated by cones (on three sides) and a wall (on the last side). Participants were tested on their ability to manipulate the ball. They were constantly motivated to provide as many solutions as possible. The examiner provided no feedback about their performance. Two-time trials were given for each skill with the following arrangements: 90 s for each trial with a 60-s resting period in between. Then, 120 s of rest were given between each dimension of fundamental movement skills. Each student was video recorded, and results were evaluated using video analysis. The score is composed of the number of solutions the student performs for each dimension of movement skills. A total score was assessed, summing score obtained in locomotor, stability, and manipulative areas [41].

#### Enjoyment

Enjoyment was recorded using the Italian version of the Physical Activity Enjoyment Scale (PACES) [42]. PACES questionnaire included 16 items classified into a 5-point bipolar Likert Scale (1 = totally disagree; 2 = disagree; 3 = not sure; 4 = agree; 5 = totally agree). A high score of positive items and a low score of negative items is related to high enjoyment. A total score can be calculated by reversing and adding the negative item score to the positive item score. The validity and reliability of the Italian version was investigated by Carraro and colleagues [42].

#### Self-perception

*Self-efficacy*. The Italian version of the Physical Self-Efficacy Scale (PSES) [43] was employed to evaluate Self-efficacy in year four elementary school students and in year three middle school students. PSES is structured into six items having a 1- to 4-point format scale. Students had to think about themselves when performing physical activity, and for each item, they had to choose the sentence that closely represents their performance. The total test is 1–24 scored. The higher the score, the higher the self-efficacy. The validity of the Italian version was investigated by Colella and colleagues [43].

*Perceived motor competence*. To measure the self-efficacy in the second-year elementary school students, the Pictorial Scale of Perceived Movement Skill Competence for Young Children (PMSC-2) [44] was used, instead of PSES. Indeed, according to previous studies [45, 46], self-efficacy, self-perception, perceived motor competence are related. PMSC-2 is structured with four graphical representations of the process of the movement. For each skill, students must think about how to perform the movement and choose images that best fit their thinking. The validity and reliability were established by Barnett and colleagues [44].

#### Video analysis

The video analysis was conducted through the System for Observing Fitness Instruction Time (SOFIT) [47] and through the Identifying the Teaching Styles (IFITS) [48]. Two out of eight lessons were randomly selected and recorded in both groups. The analysis was evaluated by some Physical Education (PE) experts using SOFIT to calculate the quantity of physical activity during each lesson and IFITS to evaluate the quality of physical activity in terms of the teaching style used. The data resulting from IFITS and SOFIT analysis were resumed and compared in the two groups as percentages. For both IFIT and SOFIT procedures, lessons were coded simultaneously by two independent observers using the same sampling pace. One of the two observers was considered the lead observer, and his data were considered for the analysis, while the second represented the reliability observer. Moreover, both observers performed the evaluation twice, distanced by a week.

Intra- and inter-rater reliability of SOFIT and IFITS was assessed using the following formula:

R% = (n° of agreement/ (n° agreement + n° disagreement)) * 100.

The intra and inter-rater reliability was considered valid if the percent of agreement (R%) overpassed the 80% [41]. The same procedure of reliability was performed for the DMA valuation.

### Procedures

**Fig 1:**
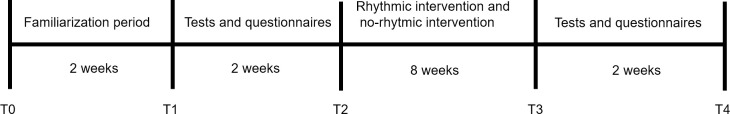
Overview of the experimental timeline.

The experimentation was led during PE classes, using the gym to perform the tests and the rhythmic and standard lessons, and the classrooms to complete the questionnaires. Anthropometric measures of height and weight were recorded to the nearest 0.1 cm with a standing stadiometer (Seca 217, Basel, Switzerland), and weight was measured to the nearest 0.1 Kg with a high-precision mechanical scale (Seca 877, Basel, Switzerland). Body mass index (BMI) was calculated as the ratio of body mass to height squared. Before testing the participants, a familiarization period was led. The two-week familiarization period included testing sessions for motor creativity and rhythmic perception. Both DMA and Mira Stambak’s tests were repeated twice for reliability. The experimental tests and the questionnaires related to enjoyment and self-perception were performed after the familiarization period. The experimental session was carried out after the tests’ performance and the completion of questionnaires. Eight rhythmic lessons, lasting one hour each, were conducted for the RI group, and eight no-rhythmic lessons were conducted for the NI group. The same PE teacher led the lessons. Two of eight lessons were recorded randomized, with a camera, and the videos were analyzed successively by a PE expert. At the end of the experimental period, tests and questionnaires were repeated. The experimental timeline is shown in Fig 1.

### Content of the lessons

The lessons were led by an expert PE teacher who worked in elementary and middle school for four years and cooperated with the University of Milan in both groups, interacting with the school’s music teacher. The 2^nd^ elementary school RI group students attended lessons focused on rhythm perception using music, listening, discovering, and inventing rhythmic sequences with gym equipment. They worked individually, in pairs, and small groups. On the other hand, the 2^nd^ elementary school NI group students attended lessons focused on topological and Euclidean space perception. They worked individually, in pairs, and in small groups as well. The 4^th^ elementary and 3^rd^ middle school RI group students attended lessons focused on rhythm in team sports such as basketball and handball, playing with music, following the rhythm of the music, or discovering rhythmic sequences in the technical skills. The 4^th^ elementary and 3^rd^ middle school NI groups attended lessons focused on team sports without taking care of rhythm perception but focusing on technical aspects, tactical strategies, and rules. Both groups worked individually, in pairs, and small groups. In the 2^nd^ elementary school classes, we used most rhythmic cadence in 4/4 and few kid’s songs as “Super simple songs for kids”. In the 4^th^ elementary and 3^rd^ middle school classes we used rhythmic cadence in 4/4 as well and we choose songs from 100 (for technical aspects as bouncing, passing the ball, basketball and handball running shots) to a maximum of 140 bpm (for the musical fitness lessons only in the 3^rd^ middle school). In 140 bpm songs we used a halved rhythm for marching or running on the spot, high knees, and toe touch, one movement every 2 quarters for squats and lunges.

### Statistical analysis

The Shapiro-Wilk normality test and kurtosis/skewness analysis [49] were performed to assess the normal distribution of the data. Data are shown as mean ± standard deviation (SD). The reliability of DMA and Mira Stambak’s tests was performed using the Intraclass Correlation Coefficient (ICC). The two-way Anova Time × Intervention (with repeated measures on time) was performed to investigate the intervention’s effects on creativity and rhythmic perceptive capacity. The significance level was set at α = 0.05 while the effect sizes were calculated as eta squared (ɳ2), using the small = 0.01, medium = 0.06, and large = 0.14 interpretation [50]. In addition, a multiple regression analysis was performed to obtain information about mediators and moderators. The mediation analysis was performed by Baron & Kenny’s method following criteria for establishing mediation in which the independent variable (IV) is the intervention, the dependent variable (DV) is the main outcome, and the mediator is the variable controlling the IV-DV relationship. The mediation models were structured by placing intervention as IV, rhythmic perceptive capacity and creativity as DV, and enjoyment as a mediator variable (MedV) together with self-perception to provide a comprehensive measure of the effect of IV on DV, Pre-Post changes for each intervention group (RI and NI) was used as DV. A dummy variable was computed to distinguish RI and NI intervention. Specifically, the dummy variable took the value 1 for data corresponding to RI and 0 for data corresponding to NI.

Additionally, a moderator analysis was employed to investigate the role of BMI and age (as ModV) in influencing the effect of IV on DV. The total, direct, and indirect effects estimate was reported using β together with 95% Confidence Intervals (CI, lower: upper limits), while the significance level was set at α = 0.05. The statistical analysis was performed using Jamovi (V. 1.6, R 4.0).

## Results

All data met the normal distribution. All tests were found reliable (DMA Locomotor: ICC = 0.945; DMA Stability: ICC = 0.936; DMA Manipulative: ICC = 0.966; DMA total score: ICC = 0.974; Mira Stambak’s test: ICC = 0.923). Table 2 shows descriptive statistics of each variable for RI And NI.

**Table 2:** Descriptive statistics for each intervention group (RI and NI).

	Variable	RI	NI
Pre	Locomotor (au)	41.9 + 7.2	41.7 + 5.3
	Stability (au)	31.8 + 6.5	30.7 + 5.2
	Manipulative (au)	36.4 + 8.7	35.4 + 7
	DMA total score (au)	110.1 + 19.8	107.8 + 13.6
	Mira Stambak (au)	32.8 + 5.2	30.4 + 6.5
	PACES (au)	69.2 + 10.7	70.7 + 9.8
	PMSC—2 (au)	31.4 + 4.7	33.6 + 5
	PSES (au)	18.7 + 3.1	19.1 + 3.8
Post	Locomotor (au)	47.7 + 5.5	41.7 + 4.9
	Stability (au)	37.4 + 9.5	31.2 + 8.9
	Manipulative (au)	44.6 + 7.4	36.2 + 7.6
	DMA total score (au)	128.7 + 18.2	109.1 + 13.8
	Mira Stambak (au)	37.7 + 1.5	29.5 + 4.9
	PACES (au)	75.4 + 4.9	65.2 + 8.3
	PMSC—2 (au)	35.6 + 3.5	31.1 + 4.4
	PSES (au)	20.4 + 3.3	17.5 + 4.2

Data are mean ± SD; RI = Rhythm Intervention; NI = No-Rhythm Intervention; DMA = divergent movement ability test; PACES = physical activity enjoyment scale; PMSC = perceived movement skill competence scale; PSES = physical self-efficacy scale.

**Fig 2:**
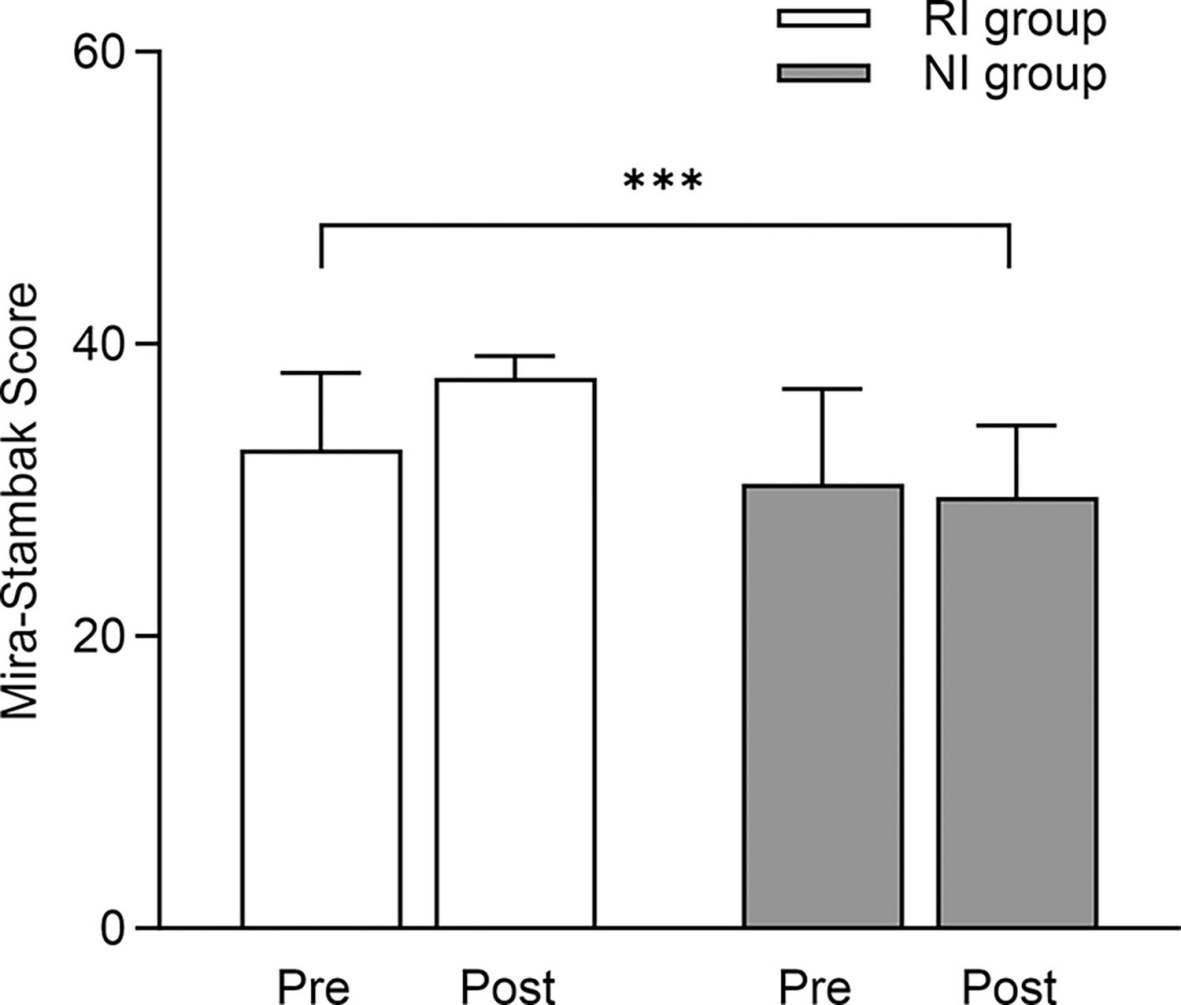
Effect of RI and NI on rhythmic perceptive capacity assessed by the Mira-Stambak score. Data are mean ± SD. ***p < 0.001 for time × intervention interaction. RI = Rhythmic Intervention. NI = No-Rhythmic Intervention.

For rhythmic perceptive capacity, a significant Time × Intervention interaction was found (p < 0.001; ɳ2 = 0.080, medium). From Pre to Post, the RI group improved better than the NI group (Fig 2).

**Fig 3:**
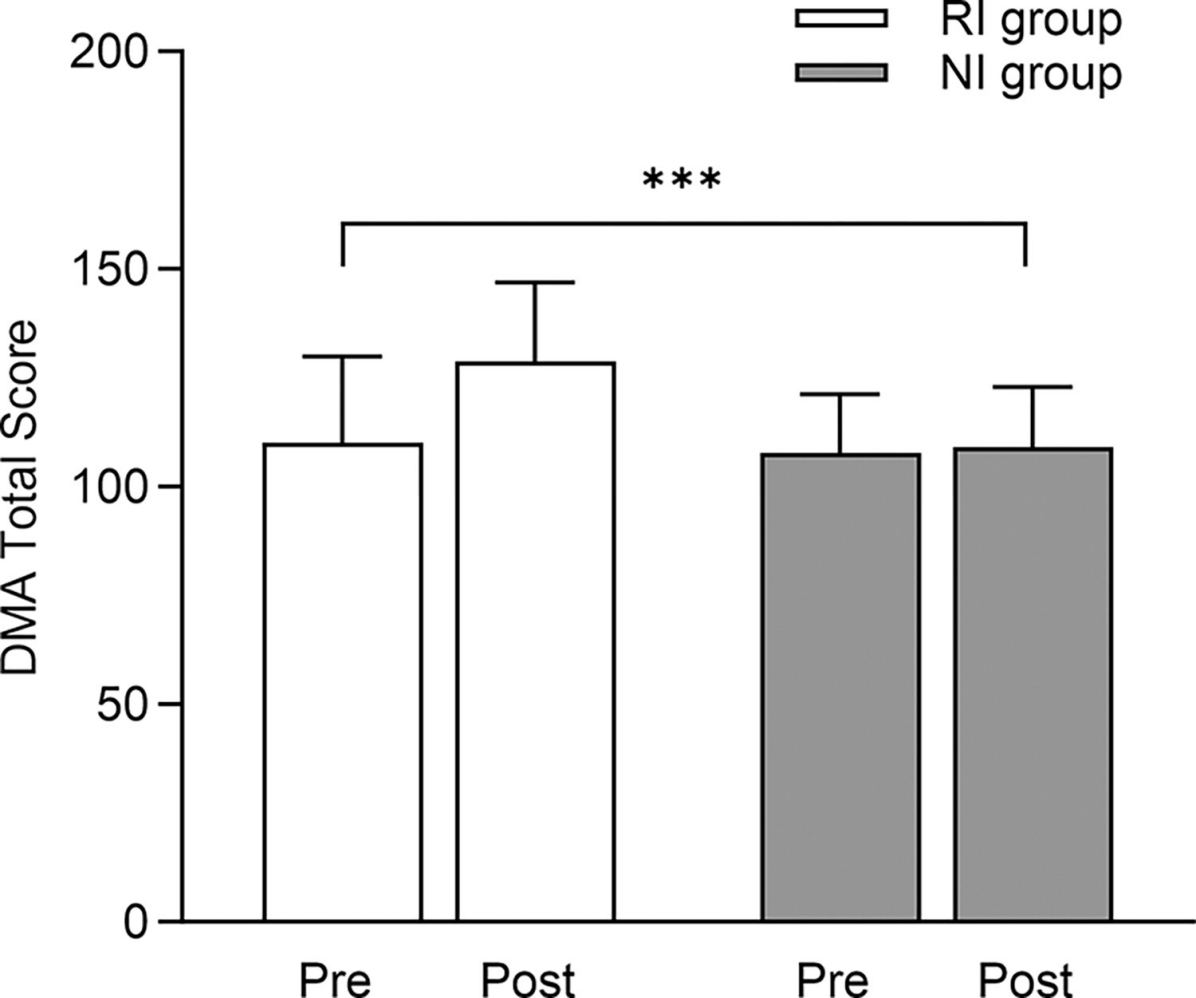
Effect of RI and NI on creativity assessed by the Divergent Movement Ability (DMA) total score. Data are mean ± SD. ***p < 0.001 for time × intervention interaction. RI = Rhythmic Intervention. NI = No-Rhythmic Intervention.

For creativity, a significant Time × Intervention interaction was found (p = 0.001; ɳ2 = 0.041, small). From Pre to Post, the RI group improved better than the NI group (Fig 3).

The first mediation analysis revealed that the intervention had a significant direct effect on rhythmic perceptive capacity (β = 4.89, CI = 1.83:7.96; p = 0.002). Conversely, no significant mediation by enjoyment was found (i.e., indirect effect; β = 1.02, CI = -0.92:2.96; p = 0.30).

Moreover, the second mediation analysis revealed that the intervention had no significant direct effect on creativity (β = 4.09, CI = -4.83:13.0; p = 0.36). However, enjoyment significantly mediated the effect of the intervention on creativity (β = 7.63, CI = 1.76:13.5; p = 0.011).

**Fig 4:**
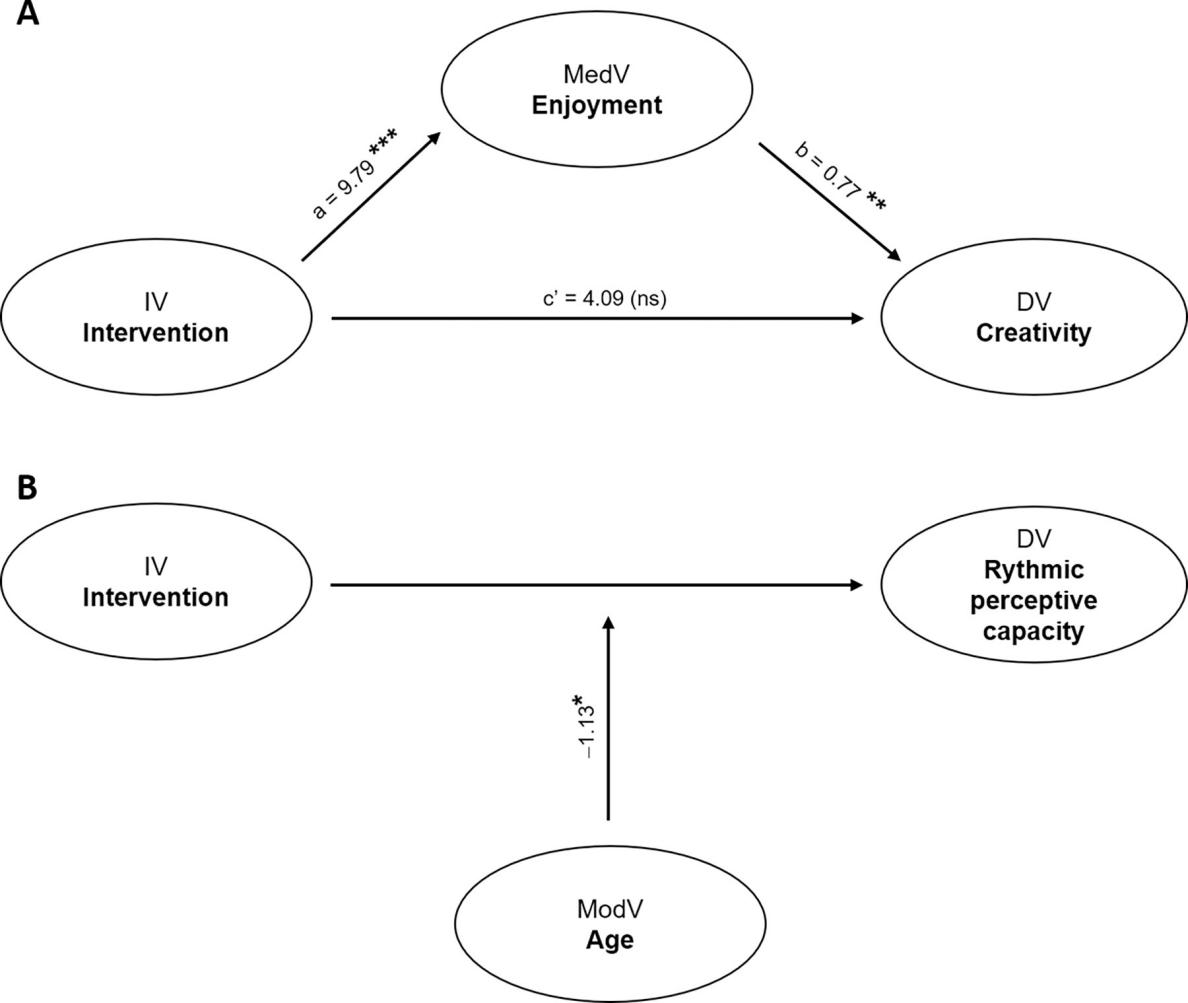
Panel A: Mediation model explaining the effect of intervention on creativity mediated by enjoyment. Panel B: Moderator model demonstrating age’s impact on the effect of the intervention on rhythmic perceptive capacity. IV: independent variable; MedV: mediation variable; ModV: moderator variable; DV: dependent variable; a: effect of IV on MedV; b: effect of MedV on DV; c’: direct effect of IV on DV controlling for MedV. **p < 0.01; ***p < 0.001; ns: non-significant.

The third mediation analysis with self-perception variables showed that no significant mediation by PSES and PMC-2 was found (i.e., indirect effect; β = -0.63, CI = -1.67:0.39; p = 0.22; β = -0.99, CI = -4.18:2.20; p = 0.54) on the effect of the intervention on rhythmic perceptive capacity. Similarly, the fourth mediation analysis with self-perception variables showed that no significant mediation by PSES and PMC-2 was found (i.e., indirect effect; β = 2.12, CI = -1.48:5.73; p = 0.24; β = 4.42, CI = -2.80:11.16; p = 0.23) on the effect of the intervention on creativity. The moderation analysis revealed a significant moderating impact of age on the relationship between intervention and rhythmic perceptive capacity (β = -1.13, CI = -2.16:-0.09; p = 0.032), but not of BMI (β = -0.62, CI = -1.54:0.30; p = 0.18). Regarding creativity, the moderation analysis revealed a non-significant moderating impact neither of age (β = -2.60, CI = -5.44:0.24; p = 0.07) nor of BMI (β = -1.41, CI = -4.13:1.30; p = 0.30) on intervention–creativity relationship. Fig 4 shows the structural model with the outcome of mediation and moderation analysis.

Both SOFIT and IFITS evaluations resulted reliable (SOFIT–Intra-rater reliability: R% = 84%, Inter-rater reliability: R% = 82%; IFITS—Intra-rater reliability: R% = 80%, Inter-rater reliability: R% = 81%). The qualitative and quantitative analysis of teaching styles are shown in Table 3.

**Table 3:** SOFIT results for RI e NI groups and for grades.

Group	Grades	Duration (min)	% MVPA	% MC	% PROM
RI	2^nd^ elementary school	57.5 ± 2.1	40.0 ± 0.2	47.7 ± 4.8	37.5 ± 3.7
NI	2^nd^ elementary school	59.0 ± 11.3	29.4 ± 11.5	53.7 ± 10.8	23.7 ± 19.5
RI	4^th^ elementary school	51.3 ± 2.4	62.3 ± 3.0	56.3 ± 3.7	32.7 ± 1.4
NI	4^th^ elementary school	47.2 ± 3.1	44.9 ± 0.6	54.3 ± 3.5	0.0 ± 0.0
RI	3^rd^ middle school	38.8 ± 4.5	62.8 ± 20.1	74.5 ± 18.1	38.6 ± 49.8
NI	3^rd^ middle school	37.0 ± 11.3	25.5 ± 3.6	65.6 ± 4.8	0.0 ± 0.0
RI mean		49.2 ± 8.9	55.0 ± 14.8	59.5 ± 14.9	25.7 ± 29.4
NI mean		47.7 ± 12.3	33.3 ± 10.6	57.9 ± 8.1	7.9 ± 15.0

RI = rhythmic intervention; NI = No-rhythmic intervention; MVPA = moderate to vigorous physical activity; MC = motor content; PROM = physical activity promotion.

IFITS’s results per group and grade are shown in Table 4.

**Table 4:** IFITS’s results per group and grade.

Style Duration	2^nd^ elementary school		4^th^ elementary school		3^rd^ middle school		Group mean	
(%)	RI	NI	RI	NI	RI	NI	RI	NI
Command	14.6 ± 10.7	4.6 ± 1.9	30.5 ± 8.4	23.5 ± 8.2	8.0±0.7	10.4 ± 14.7	17.7 ± 12	12.8 ± 11.5
Practice	16.4 ± 23.2	0.0 ± 0.0	39.9 ± 2.2	31.0 ± 7.2	47.0±9.6	28.4 ± 2.5	34.4 ± 18.2	19.8 ± 15.7
Reciprocal	10.5 ± 14.9	6.8 ± 2.2	0.9 ± 1.3	0.0 ± 0.0	6.5 ± 9.3	4.8 ± 6.7	6.0 ± 9.0	3.9 ± 4.5
Self-Check	0.0 ± 0.0	0.0 ± 0.0	0.0 ± 0.0	0.0 ± 0.0	0.0 ± 0.0	4.8 ± 6.7	0.0 ± 0.0	1.6 ± 3.9
Inclusion	0.0 ± 0.0	0.0 ± 0.0	0.0 ± 0.0	0.0 ± 0.0	0.0 ± 0.0	0.0 ± 0.0	0.0 ± 0.0	0.0 ± 0.0
Guided Discovery	0.0 ± 0.0	12.5 ± 17.7	19.8 ± 6.7	20.7 ± 2.4	10.7 ± 15.2	5.6 ± 7.9	10.2 ± 11.6	12.9 ± 11.1
Divergent	4.3 ± 6.1	15.8 ± 13.9	2.5 ± 3.6	0.0 ± 0.0	0.0 ± 0.0	2.4 ± 3.4	2.3 ± 3.7	6.0 ± 9.9
Go Beyond	0.0 ± 0.0	0.0 ± 0.0	0.0 ± 0.0	0.0 ± 0.0	0.0 ± 0.0	0.0 ± 0.0	0.0 ± 0.0	0.0 ± 0.0
Management	54.2 ± 8.6	60.3 ± 0.3	6.6 ± 1.3	24.8 ± 13	27.7 ± 14.1	43.8 ± 12.5	29.5 ± 22.6	43 ± 17.8

RI = Rhythm Intervention; NI = No-Rhythm Intervention.

## Discussion

This study in teaching physical education investigated the effect of a rhythmic approach on motor creativity and rhythmic perceptive capacity. The protocol of this research was based on system thinking and rhythms methodology. Teaching-learning and enjoyment in physical education are the main system elements that have been hypothesized to interact closely to determine direct or mediated effects on motor creativity and rhythmic perceptive capacity.

The main findings of the present study were that rhythmic teaching in physical education improved the rhythmic perceptive capacity and motor creativity in young students, which could be beneficial for other school subjects (i.e., music) [6]. Furthermore, such a teaching approach would increase the amount of moderate and vigorous physical activity during PE lessons in line with the recommendations and strategies for promoting motor practice established by the World Health Organization and other policy statements [16, 51].

The rhythmic approach let the RI group achieve more significant improvements in rhythmic perception than the NI group, which did not focus on this ability, as Mira Stambak’s test results showed. The significant improvements in the RI group’s rhythmic perception compared to the NI group may be attributed to the specific nature of the intervention [52].

Analyzing the results of the DMA, the RI group showed greater motor creativity than the NI group after the eight-week intervention, although the guided and divergent discovery styles were used more in the NI group. Our data appears to contradict existing literature [33], suggesting that a nonlinear approach is primarily conducive to developing motor creativity and creative thinking [22, 25]. Despite greater use of reproductive and linear styles, the rhythmic intervention has represented the leverage point in the context of system thinking through which it was possible to obtain substantial changes in motor creativity [6, 20].

Specific studies show how rhythm plays an important role in vital functions such as breathing and circulation and to guarantee coordinated actions in any movement [53].

Rhythm is the basis of all coordinated movement, and consequently, rhythm and movement can be efficiently combined to increase effectiveness in these interdependent domains [54].

The rhythmic abilities of children and young people encompass creative experiences, including imitation and interpretation, such as folk and social dances, singing rhythms, auditory rhythmic mental representations, and motor skill guidance [15].

By considering the relative importance of various factors in the context of system thinking, we hypothesized that utilizing an integrated multiple teaching style, which was indeed achieved in rhythmic physical education, could have led to a non-linear causal relationship with a more substantial outcome effect [22, 55].

Furthermore, greater use of practice and command styles in the RI group could allow learning better and consolidating rhythm and motor creativity skills. These observations are confirmed by the specific literature pointing out that internalization of a domain requires learning its rules and content through a large amount of information and frequency of stimuli obtainable through repeated exercises such as those guaranteed by practice and command styles [56] (Table 4). Conversely, management was less employed in the RI group compared with the NI group in every grade. This may be due to the rhythmic approach and styles that require less organizational management than a productive approach [57]. Analysis of the quantitative aspects (Table 3) of the SOFIT data showed that although the duration and motor content were similar in both groups, the RI group performed more MVPA% (Moderate to Vigorous Physical Activity%). This confirms how even this quantitative factor stimulated by the rhythmic proposition, probably due to the fast-paced nature that a rhythmic approach may require, may have contributed to a higher chance of exercising and assimilating the domain [16].

Furthermore, this data is not of secondary importance when also considering the effect of rhythmic teaching in increasing active practice (moderate and intense) during lessons with the consequent health benefits reported in the literature [16, 51, 58].

The reciprocal style primarily used in the RI group allows a continuous relationship and feedback between students. Furthermore, the student acting as an observer learns to analyze and correct his partner’s mistakes and stimulates critical thinking, which is essential in developing motor creativity. The interaction between the students determines the solicitation of social skills, making the proposals more exciting and pleasant [7, 57].

The mediation analysis revealed that enjoyment was the only mediator that had a significant impact on the relationship between motor creativity and intervention. None of the other mediators under consideration had any impact on either the relationship between intervention and motor creativity or the relationship between intervention and rhythmic perception. The observed results can be primarily attributed to the direct impact of the rhythmic intervention, particularly when combined with a specific multi-teaching styles methodology [7, 15, 22].

Durable sounds and sensations, such as those of the lullaby that mothers sing to children to make them fall asleep, determine sweet and pleasant rhythmic sensations, and reinforce the bond with their mother [59]. This example illustrates how appropriate rhythmic activities in physical education, such as songs and simple dances, can create enjoyable and satisfying experiences, thereby stimulating the desire for learning and fun in children and young people [16, 60].

Furthermore, listening to pleasant music integrated with movement helps stimulate divergent thinking, a key element of creativity [61]. However, it was impossible to consider this “music component” through the IFITS analysis because it was not contemplated as a possible type of divergent style.

By looking at the data from the perspective of the key concepts of system thinking, some effects could be delayed and made observable over a longer period [62]. This effect could occur when trying to change a complex system. Behavioral and interaction fluctuations (characterizing complex systems) may hide more possible connections between the variables and the mediators considered, making it take longer to see other possible effects [62].

Although the literature recognizes possible relationships between positive self-perception, enjoyment, and competence not only in physical education but in numerous fields of school learning [63], we believe that the construction of a body image capable of mediating the relationship between rhythm and motor creativity, being the product of variables involving multiple domains in a transversal form, could require a longer intervention time to be assimilated determining a mediation effect [64, 65].

The moderation analysis of intervention-rhythmic perception and age relationships shows a significant effect, highlighting how age and previous experience can affect rhythmic perception. Regarding motor creativity, weight appears to condition this domain. Students with a higher weight are generally older than those with a lower weight. Therefore, the age of practice could also condition motor creativity, and their expertise confirmed the relationship between age and DMA [6].

From the emerging results of this study, a multilevel model [4] based on system thinking can be characterized by three systems that, through a closed chain of causal connections, determine the effectiveness of the systemic approach. These three systems are:

- The exosystem, which is made up of the most distant level from the subjects towards whom the experimentation is directed. This system is represented by legislation, policies, and governance systems that, in a scholastic context, represent the national guidelines (ministerial guidelines) [7] that, by the support of empirical and scientific evidence, have acknowledged the important role that rhythmic education, creativity and the transversal/transdisciplinary nature of the proposals, play in the education of children and young people;
- the mesosystem, which represents an intermediate level, constituted by the cultural institutional reality of the school, made up of families, school leaders, teaching subjects, and other stakeholders. The efficacy of this intermediate level is characterized by an environment that permits the implementation of transversal educational experiments, avoiding being excessively repressive and conservative of boundaries of each discipline;
- the endosystem, which is made up of the environment in closest contact with the school children, of related teaching domain (music and physical education school subject) and its related fields (positive relationship and interaction between teachers transversally involved) [4].

This way, particularly relevant, is the teachers who direct the students’ interest, support them, and consider the students’ abilities by creating the right balance between "challenging proposals and execution skills" to promote pleasure and interest and avoid frustration by proposing tasks that are inadequate for them.

The mediation analysis revealed that enjoyment did not mediate the effect of the intervention on rhythmic perceptive capacity, suggesting that other variables (not assessed in the present study, such as attention, focus, and cognitive engagement) may play a mediating role in the relationship between the intervention and rhythmic perceptive capacity. This finding agrees with Vazou and colleagues [16].

Regarding creativity, the mediation analysis revealed that enjoyment significantly mediated the effect of the intervention on creativity. This suggests that the positive effect of the rhythm-based intervention on creativity was partially mediated by the enjoyment experienced by the students during the intervention. These findings are consistent with previous research highlighting the importance of enjoyment and its positive effect in promoting creativity [66, 67]. The intervention’s rhythmic activities and music-based elements may have elicited positive emotions and a sense of enjoyment, enhancing the students’ creative thinking and expression. Overall, this emphasizes the teacher’s role in conducting physical education programs (like rhythmic-based interventions) utilizing strategies promoting enjoyment [68].

The moderation analysis showed that age had a significant moderating impact on the relationship between the intervention and rhythmic perceptive capacity, indicating that the effect of the intervention on rhythmic perceptive capacity varied depending on the age of the students. This finding suggests that younger students may benefit more from rhythm-based interventions to improve their rhythmic perceptive abilities. However, no significant moderating impact of BMI was found, indicating that BMI did not influence the effectiveness of the intervention on rhythmic perceptive capacity. These findings are consistent with a recent study showing age-related differences in rhythm perception [6]. Younger students’ developmental stage and cognitive abilities may make them more receptive to rhythm-based activities and interventions.

Regarding creativity, the moderation analysis revealed a non-significant moderating impact of age and BMI on the intervention-creativity relationship. This indicates that neither age nor BMI significantly moderated the intervention’s and creativity’s relationship. These findings suggest that the rhythm-based intervention positively affected creativity across different age groups and BMI categories. This aligns with research showing the universal benefits of music and movement interventions on creativity in diverse populations [69].

## Limitations of this study

There is a paucity of literature on the relationships between rhythmic-motor teaching, motor creativity, teaching styles, children’s motor engagement during the lessons, enjoyment, and self-perception. It is therefore essential to recognize that the scholastic thinking system could represent the foundation of future studies to provide objective data on the effectiveness of the framework of the ecological model in physical education [6].

Data of our model include a small number of participants for a clear analysis that considers differences in age, gender, and other mediators and moderators considered self-perception or weight. In particular, as for self-perception, we used two types of questionnaires to measure self-efficacy and perceived motor competence (due to the lack of a single validated instrument capable of evaluating this component in children in the first elementary classes and the young in the last years of middle school), the small number further reduce the representativeness of the outcomes.

### Practical applications

System thinking allowed us to highlight two fundamental points of rhythmic teaching (leverage and resistance points) to improve motor creativity. Thanks to the particularity of these domains, the transversality between physical education and rhythmic-musical education is a leverage point that can lead to effective results. A little skilled teacher and an unfavorable environmental reality could represent a resistance point. To avoid this, a competent teacher should have the ability to create, through a varied and engaging rhythmic multi-teaching approach, adequate relationships and enjoyment with the students to whom he relates (proposals adequate to the age and subjects’ characteristics, suiting what they can and know to do). Without these conditions, the resistance points compromise the project’s possibility. An environmental reality (educational managers, music teachers, parents, educational and ministerial programs) should be based, above all, on the possibility of encouraging overcoming the educational boundaries of the subjects involved in the project (possibility of blending physical education with other subjects).

## Conclusions

This study emphasizes the relationship between rhythms and motor creativity and the importance of enjoyment in engaging during these lessons’ typology. The results of the present study, which also considered teaching styles and the type of motor practice, provide new insights for school teaching and support system thinking as a way to understand how all variables involved in the learning process can favor or hinder learning itself. Identifying, distinguishing, and analyzing the relationship between what is taught and what is produced represents a reflective practice confirming the adherence of our study with the reference frameworks setting themselves to an obliquity (to consider organizational, methodological, and environmental factors), and making PE teaching even more helpful and cutting edge.

## Supporting information

S1 File (XLSX)
